# Molecular fingerprints of cardiovascular toxicities of immune checkpoint inhibitors

**DOI:** 10.1007/s00395-024-01068-8

**Published:** 2024-07-17

**Authors:** Tamás G. Gergely, Zsófia D. Drobni, Nabil V. Sayour, Péter Ferdinandy, Zoltán V. Varga

**Affiliations:** 1 Center for Pharmacology and Drug Research & Development, Department of Pharmacology and Pharmacotherapy, Semmelweis University, Budapest, Hungary; 2 HCEMM-SU Cardiometabolic Immunology Research Group, Budapest, Hungary; 3 MTA-SE Momentum Cardio-Oncology and Cardioimmunology Research Group, Budapest, Hungary; 4 Heart and Vascular Center, Semmelweis University, Budapest, Hungary; 5 Pharmahungary Group, Szeged, Hungary

**Keywords:** Cardiotoxicity, Immunotherapy, Autoimmune, Immune-related adverse event

## Abstract

Immune checkpoint inhibitors (ICIs) have revolutionized cancer therapy by unleashing the power of the immune system against malignant cells. However, their use is associated with a spectrum of adverse effects, including cardiovascular complications, which can pose significant clinical challenges. Several mechanisms contribute to cardiovascular toxicity associated with ICIs. First, the dysregulation of immune checkpoints, such as cytotoxic T-lymphocyte-associated protein 4 (CTLA-4) and programmed cell death protein-1 (PD-1) and its ligand (PD-L1), and molecular mimicry with cardiac autoantigens, leads to immune-related adverse events, including myocarditis and vasculitis. These events result from the aberrant activation of T cells against self-antigens within the myocardium or vascular endothelium. Second, the disruption of immune homeostasis by ICIs can lead to autoimmune-mediated inflammation of cardiac tissues, manifesting as cardiac dysfunction and heart failure, arrhythmias, or pericarditis. Furthermore, the upregulation of inflammatory cytokines, particularly tumor necrosis factor-alpha, interferon-γ, interleukin-1β, interleukin-6, and interleukin-17 contributes to cardiac and endothelial dysfunction, plaque destabilization, and thrombosis, exacerbating cardiovascular risk on the long term. Understanding the intricate mechanisms of cardiovascular side effects induced by ICIs is crucial for optimizing patient care and to ensure the safe and effective integration of immunotherapy into a broader range of cancer treatment protocols. The clinical implications of these mechanisms underscore the importance of vigilant monitoring and early detection of cardiovascular toxicity in patients receiving ICIs. Future use of these key pathological mediators as biomarkers may aid in prompt diagnosis of cardiotoxicity and will allow timely interventions.

## Introduction

Immune checkpoint molecules, such as programmed cell death protein-1 (PD-1) and its ligand (PD-L1), cytotoxic T-lymphocyte-associated protein 4 (CTLA-4), or lymphocyte-activation gene-3 (LAG-3), are physiological regulators of immune activation. Immune checkpoint inhibitors (ICIs), targeting these molecules, have revolutionized cancer treatment by enhancing the immune system’s ability to recognize and eventually kill malignant cancer cells. However, this immunomodulatory therapy is associated with a range of immune-related adverse events (irAEs) [97], including cardiovascular complications. Understanding the types, occurrence, and pathomechanisms of these cardiovascular side effects is crucial for finding new treatment options, optimizing patient care, and ensuring the safe use of ICIs in oncology practice [18, 112].

**Fig. 1:**
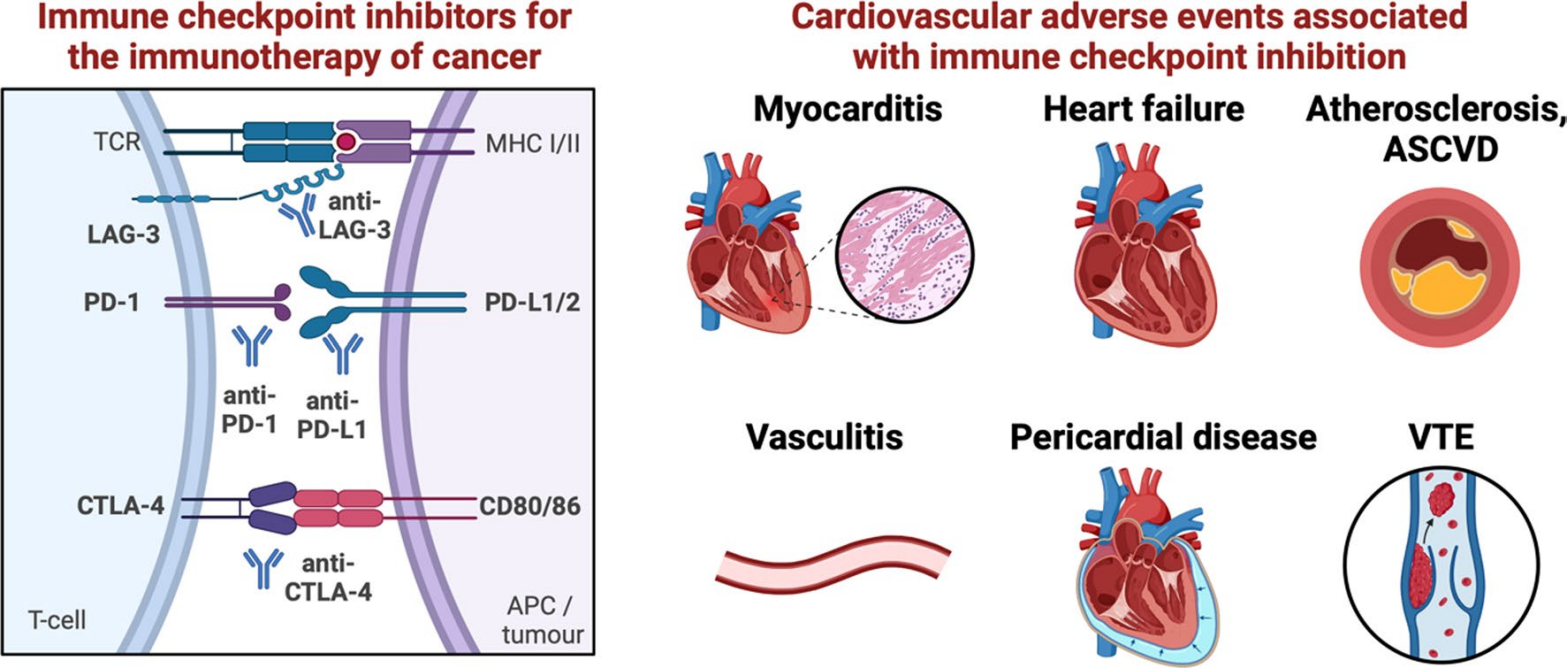
Immune checkpoint inhibition in cancer therapy and its cardiovascular adverse effects. (Left) Inhibition of immune checkpoints via monoclonal antibodies is an effective pharmacological strategy for anti-cancer treatment. (Right) Immune checkpoint inhibition is associated with immune-related adverse events, including cardiovascular side effects. These include myocarditis, cardiac dysfunction or heart failure, progression of atherosclerosis and atherosclerotic cardiovascular disease (ASCVD), vasculitis, pericardial disease, and venous thromboembolism (VTE). LAG-3: lymphocyte-activation gene-3, PD-1: programmed cell death protein-1, PD-L1: programmed death ligand-1, CTLA-4: cytotoxic T-lymphocyte-associated protein 4. Created with Biorender.com

Cardiovascular adverse events may involve any part of the heart and the vasculature; therefore, now a full spectrum of cardiovascular toxicities are recognized (Fig. 1) [77]. Myocarditis, characterized by inflammation of the myocardium, is a serious and potentially life-threatening cardiovascular side effect of ICIs [58]. It can present with symptoms such as chest pain, dyspnea, palpitations, and fatigue. Myocarditis associated with ICIs often manifests as acute heart failure, arrhythmias, or cardiogenic shock [86]. Prompt recognition and management are essential to prevent cardiac damage and improve outcomes. Beyond myocarditis, other cardiovascular irAEs have been reported such as arrhythmias, left ventricular (LV) dysfunction, Takotsubo syndrome, pericarditis and pericardial effusion, vasculitis, accelerated atherosclerosis progression, acute coronary syndrome and thromboembolic events, many of them appearing without evidence of myocarditis [9, 40]. Cardiovascular side effects upon ICI treatment were initially considered relatively uncommon compared to other immune-related adverse events (such as endocrine and gastrointestinal side effects); however, their incidence varies depending on the type of inhibitor, combination therapies, and patient characteristics. The whole spectrum of cardiovascular irAEs has been assessed in recent meta-analyses, reporting the rate of cardiovascular toxicities to be 3–7% [25, 103]. However, in primary studies focusing on assessing cardiovascular event rates, reporting of cardiovascular side effects is higher [30, 62].

The pathomechanism of ICI-induced cardiovascular side effects is complex, involving disruption of immune tolerance mechanisms, inflammatory tissue injury, endothelial dysfunction, and may potentially be related to genetic predisposing factors, that are yet to be explored. In the present review, we aim to summarize the current understanding of the pathomechanisms of ICI-induced cardiovascular toxicities in the hope of finding key shared mechanisms as therapeutic targets, allowing parallel reduction of a wider spectrum of cardiovascular side effects.

## Immune checkpoint inhibitor-induced myocarditis

### Clinical relevance

**Fig. 2:**
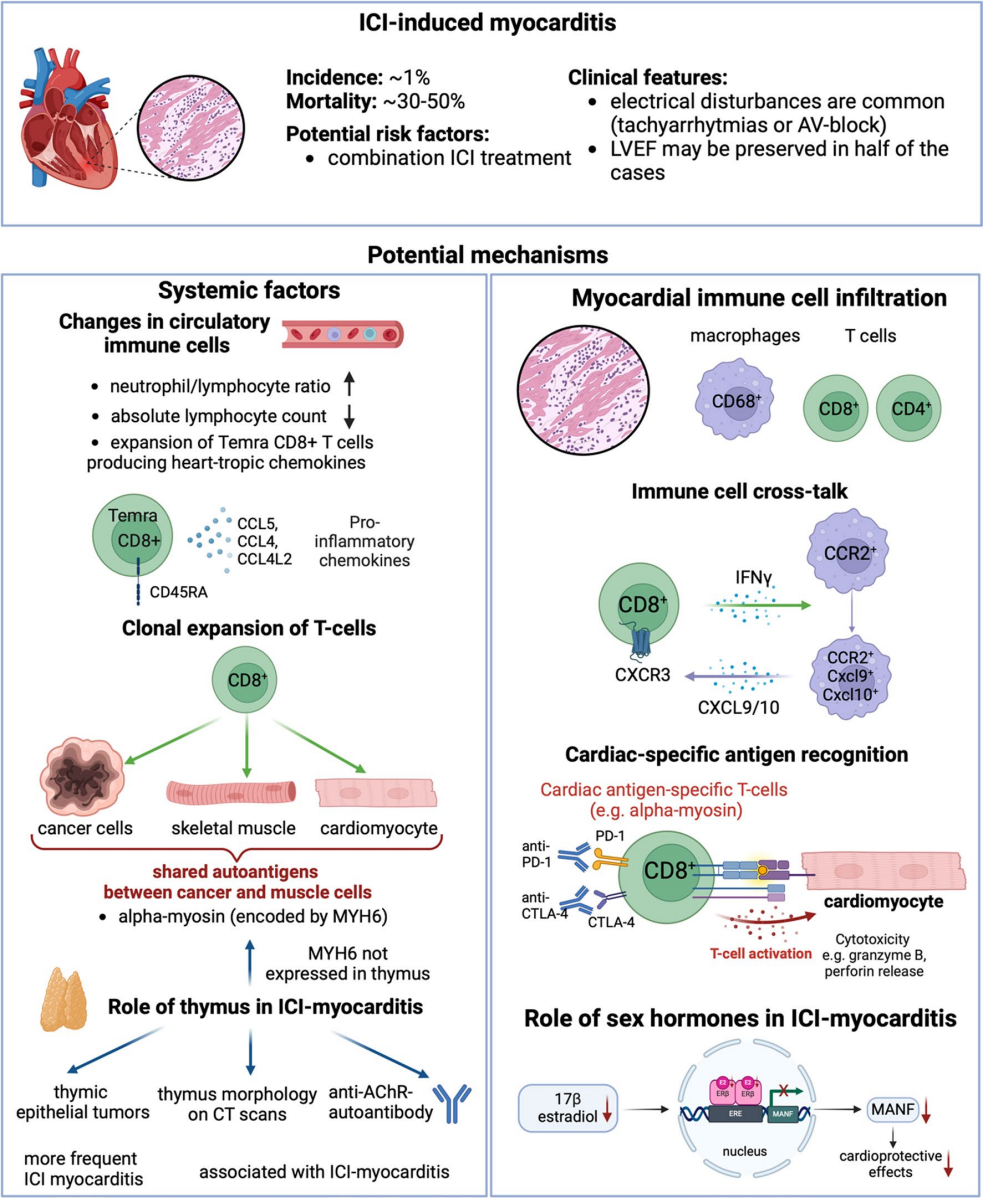
Immune checkpoint inhibitor (ICI)-induced myocarditis. ICI myocarditis is a rare, but fatal cardiac adverse event. Potential mechanisms include systemic factors, such as changes in circulatory immune cells, clonal T-cell expansion due to shared antigens between the tumor cells, cardiomyocytes, and skeletal muscle cells, and the central role of the thymus. Local alterations in the myocardium include immune cell infiltration, crosstalk between T cells and macrophages, and cardiac-specific antigen recognition by T cells, while changes in estrogen hormone levels may alter local cardioprotective signaling. CCL5: chemokine (C–C motif) ligand 5, CCL4: chemokine (C–C motif) ligand 4, CCL4L2: chemokine (C–C motif) ligand 4 like 2, MYH6: myosin heavy chain 6, AChR: acetylcholine receptor, CCR2: C–C chemokine receptor type 2, CXCR3: C-X-C motif chemokine receptor 3, CXCL9/10: C-X-C motif chemokine ligand 9/10, PD-1: programmed cell death protein-1, CTLA-4: cytotoxic T-lymphocyte-associated protein 4, MANF: mesencephalic astrocyte-derived neurotrophic factor. Created with Biorender.com

ICI-induced myocarditis was the first cardiovascular adverse effect of ICI treatment that gained widespread attention following early case reports with fulminant presentation [58]. Although it is a relatively rare complication, occurring in about 1% of cases, high mortality is seen in patients [81]. ICI myocarditis frequently presents with arrhythmias [105], while left ventricular ejection fraction is preserved in about half of the cases [81]. Risk factors for myocarditis development after ICI treatment are not fully understood, with combination ICI treatment showing the strongest association [105]. As the number of cancer patients with ICI treatment increases rapidly, ICI myocarditis has been investigated thoroughly in the past years [45, 86, 91, 115], including its diagnosis and screening [69] and potential treatment options [104]. Deeper insights into its molecular mechanisms are needed to understand better and treat this devastating adverse effect (Fig. 2).

### Potential Mechanisms

Since the observations of severe myocarditis induced by ICI treatment in patients, numerous studies aimed to discover the mechanisms to understand patients at risk and to develop potential treatment options. An early case report described the infiltration of CD68^+^ macrophages and CD4^+^ and CD8^+^ T cells into the myocardium of patients with ICI myocarditis [58], whereas in another case report, a predominance of CD8^+^ T cells was seen in the myocardium, with a decrease of Foxp3^+^ T_reg_ cells [64]. Further histopathological characterization revealed the wide spectrum of the grade of inflammation seen in the myocardium after ICI treatment through endomyocardial biopsy [92]. In another pathological study, an increased ratio of CD68^+^ macrophages to CD3^+^ T cells was seen in higher grade forms of ICI myocarditis [17].

Similarly, in preclinical models of ICI myocarditis, myocardial immune cell infiltration was observed. Anti-PD-1 antibody treatment in A/J mice resulted in increased myocardial infiltration of CD4^+^ and CD8^+^ T cells, as well as monocytes/macrophages and natural killer cells [120]. In this study, T-cell populations showed an increased number of effector cells and fewer naïve and memory cells compared to controls [120]. In autoimmune-prone mice (Murphy Roths Large, MRL), loss of PD-1 resulted in spontaneous myocarditis with an increased number of central memory CD4^+^, T_reg_, and effector memory CD8^+^ T cells in the myocardium [126]. Within the T-cell subsets, CD8^+^ T cells appear to play a pivotal role in fulminant myocarditis development associated with immune checkpoint inhibition: in a genetic model of *Ctla4*^+/−^ *Pdcd1*^*−/−*^ mice, immune infiltrates of CD8^+^ T cells in the myocardium were observed with increased mortality compared to littermate controls [117]. *Axelrod *et al*.* showed that depletion of CD8^+^ T cells via monoclonal antibodies, but not depletion of CD4^+^ T-cells, improved survival in this model [8]. Nevertheless, recently central memory CD4^+^ T cells have also been shown to have a protective role against ICI myocarditis [123].

Moreover, macrophages are increasingly recognized to contribute to the development of ICI myocarditis. In the established genetic mice model (*Ctla4*^+/−^ *Pdcd1*^*−/−*^*)* of myocarditis, an expansion of CCR2^+^ macrophages was seen with high expression of CXCL9 and CXCL10 among other pro-inflammatory factors, whereas a similar expanded macrophage population was observed in myocardial tissues of patients with ICI myocarditis as well [79]. Communication between T cells and the expanded macrophage population was predicted in silico to occur via the IFNγ and CXCR3 pathways, whereas inhibition of IFNγ experimentally attenuated macrophage expansion and myocarditis development, further suggesting the pathological role of macrophages in ICI myocarditis. Similarly, the expansion of CCR2^+^ CXCL9/10^+^ macrophages, along with CXCR3^hi^ CD8^+^ T cells, was seen in a preclinical model of myocarditis in MRL/Pdcd1^−/−^ mice [53]. In this study, inhibition of CXCR3 attenuated T-cell infiltration into the myocardium via inhibiting T-cell migration toward macrophages, showing the vital role of the T-cell–macrophage cross-talk in myocarditis development.

While myocardial infiltration of immune cells, particularly CD8^+^ T cells, is necessary for ICI myocarditis, questions remain about the triggering factors. Mice prone to autoimmunity (e.g., MRL mice [53]) or MHC-deficient mice with human HLA-DQ8 develop severe myocarditis after ICI treatment [100]. Moreover, mice with experimental autoimmune myocarditis (EAM) induced before anti-PD-1 treatment showed a higher grade of myocardial inflammation, while concomitant induction of EAM and administration of anti-PD-1 antibodies did not result in exacerbated myocarditis, suggesting that prior autoimmune factors might play a role in triggering ICI-induced myocarditis [113]. In the early case study by *Johnson *et al*.* [58], T-cell clones were investigated in two patients with ICI myocarditis. Interestingly, the most abundant T-cell clones in the heart were also found in the tumor and the skeletal muscle (either after ICI therapy or before and after ICI therapy as well). These findings suggest a potential shared antigen or epitope between the myocardium and the tumor, triggering clonal expansion of T cells that infiltrate into both tissues. In accordance with the mechanistic role of autoantigens in ICI myocarditis, two studies identified α-myosin (encoded by MYH6 gene) as a self-antigen targeted by CD8^+^ T cells in ICI myocarditis [8, 120]. Interestingly, MYH6 is not expressed in the thymus in mice and humans [8], thus autoreactive T cells against α-myosin might escape the thymic negative selection. Accordingly, in the study by *Won *et al*.*, cardiac-myosin-specific T cells were found in the heart, mediastinal lymph nodes, and spleen of naïve mice expressing high levels of PD-1. Thus, inhibition of the co-inhibitory PD-1 pathway via monoclonal antibodies may unleash these autoreactive T cells and initiate an immune response against the myocardium. Moreover, cardiac antigens, such as MYH6, are frequently expressed by the mutated tumor cells [8]. Nevertheless, many patients with MYH6-expressing tumors do not develop myocarditis after ICI therapy, thus suggesting that the mechanism may be more complex and that further risk factors may play a role. Besides expression in tumor cells, other possible ways exist for the development and activation of MYH6-autoreactive T cells. For example, myosin-peptide mimics can be derived from gut bacteria, which may prime Th17-type cells and induce an autoimmune reaction against the myocardium [42]. Thus, differences in the microbiome of patients may contribute to the susceptibility to ICI myocarditis.

Besides myocardial infiltration and expansion of immune cells, circulating factors have been identified in association with ICI myocarditis as well. ICI myocarditis was associated with a decreased absolute lymphocyte count and increased neutrophil-to-lymphocyte ratio [29]. In a multi-omics analysis of peripheral blood mononuclear cells (PBMCs) from patients with ICI myocarditis, an expansion of the cytotoxic Temra CD8^+^ T cells (re-expressing CD45RA) was found compared to controls, with increased expression of pro-inflammatory chemokines (e.g., CCL5, CCL4, CCL4L2). In another study investigating PBMCs via single-cell RNA sequencing, expansion of monocytes was observed in patients with ICI myocarditis, with increased expression of S100A protein family members [76]. Elevated levels of circulating cytokines have also been associated with ICI myocarditis; however, their prognostic role is not established currently [4].

Furthermore, the co-occurrence of myocarditis and myositis, as well as myasthenia-like symptoms has been reported in case studies after ICI treatment [5]. This finding points to a potential involvement of the thymus in ICI cardiotoxicity, which was recently investigated in a pharmacovigilance, biomarker-, and imaging-based study [35]. The authors found that ICI treatment of thymic epithelial tumors was more frequently associated with ICI-related myocardial and skeletal-muscle toxicities, and presented with increased severity compared to the treatment of other tumor types. Moreover, morphological characteristics of the thymus on CT scans or the presence of anti-acetylcholine-receptor antibodies associated with ICI myocarditis suggest the role of enhanced thymic activity in the pathomechanism of ICI cardiotoxicity.

Moreover, in the study by *Zhang *et al*.,* combination antibody treatment with anti-PD-1 and anti-CTLA-4 resulted in myocarditis development, associated with decreased levels of 17-β-estradiol in female mice treated with ICIs [125]. Estrogen receptor-β signaling was found to be necessary to maintain mesencephalic astrocyte–derived neurotrophic factor (MANF) transcription, whereas depletion of MANF worsened ICI myocarditis. Pharmacologically, treatment with an estrogen receptor β agonist attenuated myocarditis development.

## Immune checkpoint inhibitors and cardiac dysfunction

### Clinical relevance

Heart failure is highly prevalent in cancer patients, which may be due to shared risk factors and mechanisms and the use of cardiotoxic anti-cancer therapies [107]. Cardiac dysfunction or heart failure after ICI treatment is a potential adverse event that may occur without concomitant fulminant myocarditis, defined in the 2022 European Society of Cardiology (ESC) Guidelines on Cardio-oncology as “non-inflammatory heart failure” [77]. Heart failure has been shown in case reports to occur after ICI therapy [49], while a pharmacovigilance study identified heart failure as a late adverse event after ICI use [26]. The incidence of cardiac dysfunction or heart failure after ICI treatment in clinical studies varies greatly [40]. Nevertheless, in a large meta-analysis of 48 RCTs [25], as well as in a nationwide Danish study with ICI use [30], increased occurrence of heart failure has been associated with ICI therapies in cancer patients. In a retrospective study, *Laenens *et al*.* found that major adverse cardiac events occurred in 10.3% of patients during a median 13 months of follow-up, of which the majority (69.6%) were heart failure events [62]. Upon further investigation, most of the heart failure presentations were with preserved ejection fraction (HFpEF, 43.8%), while asymptomatic left ventricular dysfunction (31.3%), heart failure with reduced ejection (HFrEF, 18.8%) or Takotsubo syndrome (6.3%) occurred in the rest of the cases.

### Potential Mechanisms

**Fig. 3:**
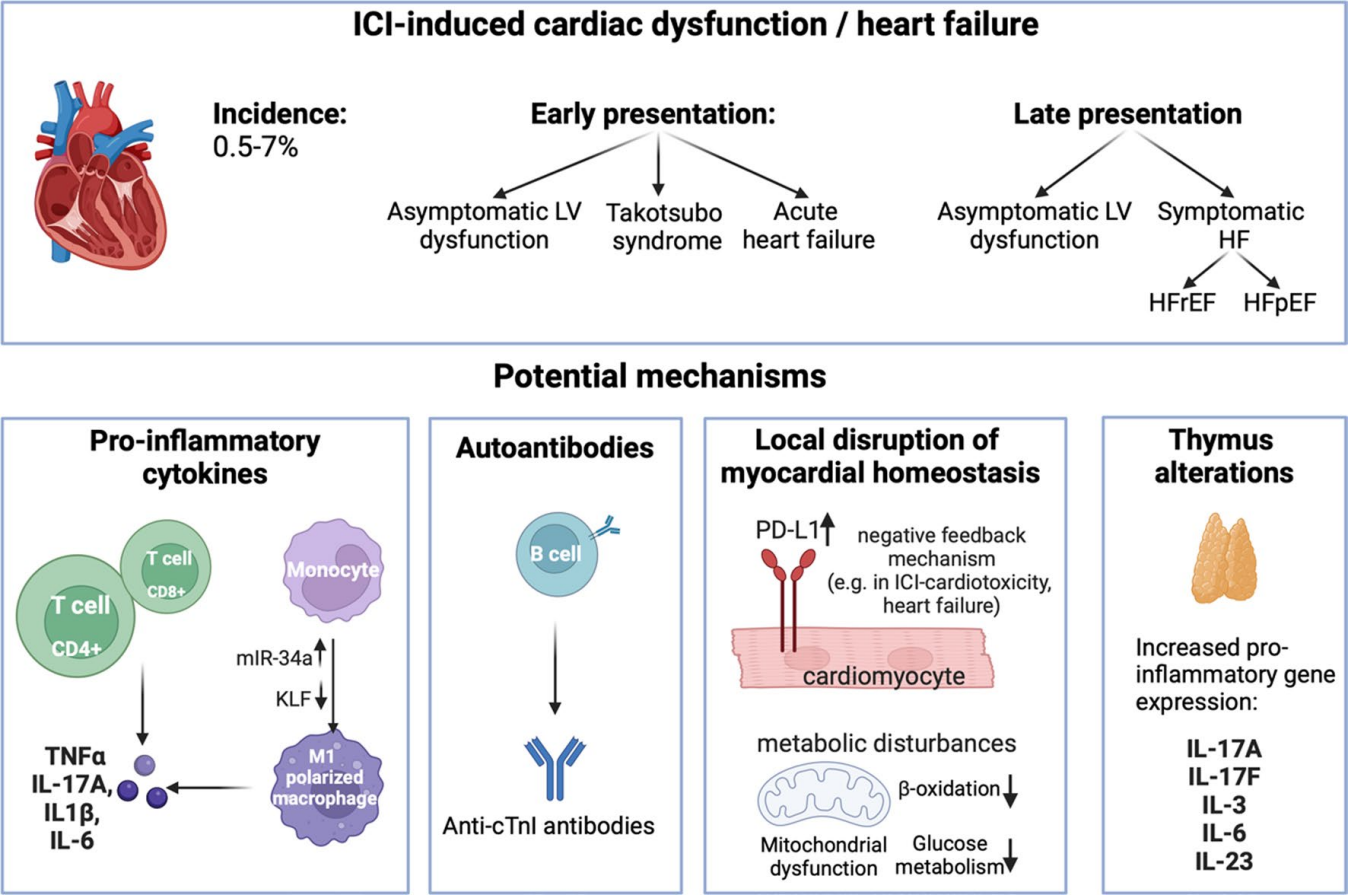
Immune checkpoint inhibitors and cardiac dysfunction or heart failure. Cardiac dysfunction can occur as an early or late adverse event after ICI treatment, with various presentations, including asymptomatic left ventricular (LV) dysfunction, Takotsubo syndrome, acute heart failure (HF), or chronic HF including HF with reduced ejection fraction (HFrEF) or with preserved ejection fraction (HFpEF). Potential mechanisms include pro-inflammatory cytokine release, autoantibodies, disruption of myocardial homeostasis and the role of the thymus. TNFα: tumor necrosis factor-alpha, IL-17: interleukin-17, IL-1β: interleukin-1β, IL-6: interleukin-6, cTnI: cardiac troponin I, PD-L1: PD-L1: programmed death ligand-1, IL-3: interleukin-3, IL-23: interleukin-23. Created with Biorender.com

While preclinical models of ICI myocarditis consistently show the vital involvement of CD8^+^ T cells and fulminant inflammation in the myocardial tissue, several models with ICI treatment report a milder response, with smoldering inflammatory changes and a consistent decline in cardiac function. Interestingly, in mice with fulminant myocarditis, cardiac function was shown to be preserved [117], thus suggesting that, at least in part, different mechanisms may play a role (Fig. 3). The association of immune checkpoint inhibitors with cardiac dysfunction or heart failure development suggests an important physiological role of immune checkpoint molecules in myocardial homeostasis and heart failure development, which has been reviewed previously [40].

Early cardiac dysfunction after anti-PD-1 treatment has been shown in preclinical models to be associated with mild inflammatory changes in the myocardium with the involvement of pro-inflammatory cytokines, e.g., TNFα [83], or interleukin-17A [41]. In line with this, in another study, mice treated with pembrolizumab (a PD-1 inhibitor) showed a transient increase in IL-17A cytokine levels after 1 week of treatment, followed by a gradual decline in systolic cardiac function [31]. Moreover, in this study, pembrolizumab induced early coronary endothelial and diastolic dysfunction. In a further preclinical study, short-term treatment with either anti-PD-1 or anti-CTLA-4 antibodies in C57BL/6 J mice resulted in early cardiac dysfunction, assessed by radial or longitudinal strain [98]. Myocardial pro-inflammatory cytokine production (e.g., IL-1α and -1β, IL-2, IL-6, IL-17A, IFNγ, and TNFα) was increased, while anti-inflammatory cytokines were decreased (e.g., IL-4 and IL-10). Moreover, in vitro treatment of cardiomyocytes with nivolumab or ipilimumab (a CTLA-4 inhibitor) increased NLRP3 expression in cardiomyocytes [99].

In preclinical models of ICI-induced cardiac dysfunction, a more pronounced CD4^+^ T-cell involvement was seen: in an in vitro study, treatment with nivolumab (a PD-1 inhibitor) in a co-culture system of human embryonic stem cell-derived cardiomyocytes with CD4^+^ T cells increased IFNγ production, while this effect was not seen in co-cultures with CD8^+^ T cells [111]. Moreover, in mice treated with pembrolizumab and subsequently developing cardiac and endothelial dysfunction [31], an early increase in myocardial CD4^+^ numbers was seen, while CD8^+^ T-cell numbers were not altered. In our group’s study, co-treatment with anti-PD-1 and a depleting anti-CD4 antibody mitigated cardiac dysfunction which was observed with anti-PD-1 treatment alone [41]. Moreover, besides T-cell involvement, macrophages have also been associated with declining cardiac function after ICI treatment. In mice treated with PD-1 inhibitors, cardiac dysfunction was associated with an induced M1-like polarization of macrophages through activation of miR-34a–Krüppel-like factor 4 signaling, while inhibition of M1-like polarization prevented the impairment of cardiac function [121].

Interestingly, in our group’s study with anti-PD-1 treatment in mice, the role of the thymus has also been suggested in ICI-induced cardiac dysfunction [41]. In our experiments, increased expression of pro-inflammatory cytokines, e.g., IL-3, IL-6, IL-17A, IL-17F, and IL-23, was seen in thymic tissues in anti-PD-1-treated C57BL/6 J mice, compared to isotype control-treated mice, while anti-inflammatory cytokine expression (e.g., IL-10) was not altered. Moreover, in BALB/c mice (with a more pronounced Th2-type immune response), the cytokine alterations were more balanced, with the concomitant increase of IL-10 anti-inflammatory cytokine expression in addition to the slight increase of pro-inflammatory cytokines. Interestingly, in this model, early cardiac dysfunction was not visible after anti-PD-1 treatment. In summary, the activation of the thymus after ICI treatment showed an important role in developing cardiac dysfunction. This finding, together with the role of thymic alterations seen in ICI myocarditis, suggests a potential central role of the thymus in mediating ICI cardiotoxicities.

## Immune checkpoint inhibitors and atherosclerosis

### Clinical relevance

Many studies have highlighted the significance of immune checkpoint pathways in the development of atherosclerosis. In patients with melanoma, the effect of ICIs on atherosclerotic plaque volumes was evaluated using routine contrast-enhanced computed tomography (CT) images. Aortic plaque volumes were quantified and compared from before to after ICI start. Total and non-calcified plaque volumes showed a greater progression rate after ICI therapy, increasing from 2.1% per year before ICI initiation to 6.7% per year after ICI initiation [27]. Similar results were found in patients with lung cancer receiving ICI therapy, where the progression rate for non-calcified plaque volume was seven times higher in those receiving ICI, compared to control lung cancer patients who received other cancer therapies (11.2% vs. 1.6% per year, *p* = 0.001). In a multivariate model including traditional cardiovascular risk factors, ICI use was associated with a more substantial progression of non-calcified plaque volume. In addition, a small subgroup receiving dual-ICI therapy showed greater plaque progression [28].

Meta-analyses and larger single-center retrospective studies have shown an increase in atherosclerosis-related cardiovascular events in patients receiving ICI therapy [110]. A pooled analysis of 59 oncological trials involving 21,664 patients showed a 35% increase in coronary ischemia over 6 months of follow-up among patients on ICI therapy compared with those on cytotoxic chemotherapies [7]. In a single-center registry of 3326 ICI-treated patients with any type of malignancy, myocardial infarction and stroke occurred in 7% and 7% of the patients, respectively [90]. Another single-center retrospective study of 2842 patients by *Drobni *et al*.* reported a 4.2% incidence of a combined atherosclerotic cardiovascular endpoint, including myocardial infarction, coronary revascularization, and ischemic stroke. These combined events were three times higher after starting ICI therapy (HR 3.3 [95% CI 2.0–5.5]) [27].

Increasing evidence underscores the potential influence of ICIs on both atherosclerosis and atherosclerotic cardiovascular events in cancer patients. Baseline atherosclerosis is prevalent among cancer patients, with around 45–75% having subclinical atherosclerosis [23]. The administration of ICI therapy correlates with a heightened likelihood of atherosclerotic cardiovascular events, such as myocardial infarction, stroke, and peripheral arterial disease, possibly driven by accelerated atherosclerosis [108]. As ICI approvals extend, particularly to adjuvant and neoadjuvant care, and patient survival rates on ICIs rise, it is crucial to weigh the potential cardiovascular event risk in this population. Although not fully understood, the pathophysiological process underlying ICI-induced atherosclerosis could be linked to inflammation and immune dysregulation [110].

### Potential mechanisms

**Fig. 4:**
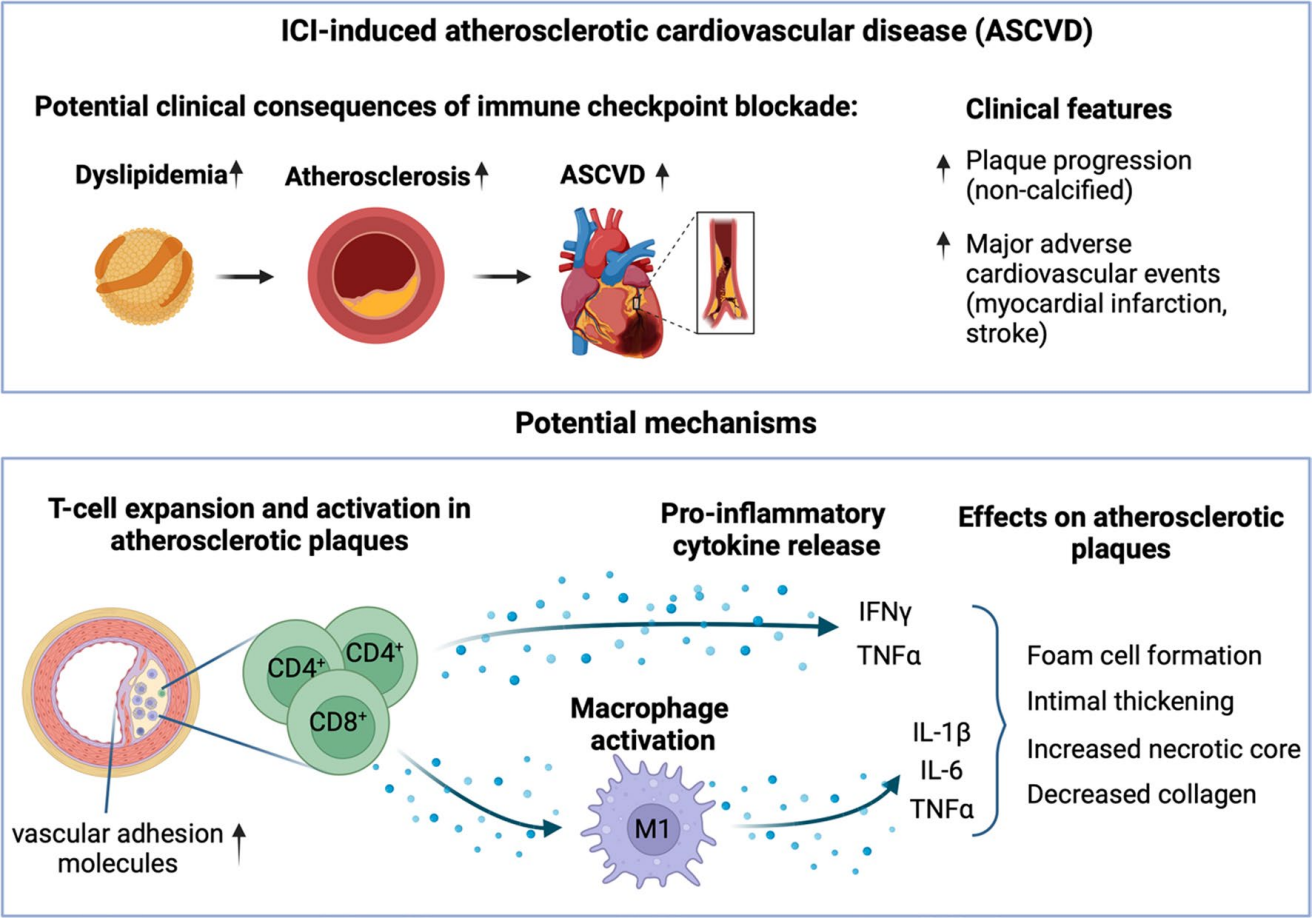
Immune checkpoint inhibitor-induced atherosclerotic cardiovascular disease (ASCVD). Immune checkpoint inhibition may contribute to ASCVD by promoting plaque progression or by increasing dyslipidemia. Overall, an increase in major adverse cardiovascular events is observed after ICI therapy. Potential mechanisms include T-cell expansion and activation in the atherosclerotic plaques, crosstalk between T cells and macrophages leading to macrophage activation, and the release of pro-inflammatory cytokines. Inflammation in the plaque results in foam cell formation, intimal thickening, increased necrotic core and decreased collagen content. IFNγ: interferon-γ, TNFα: tumor necrosis factor alpha, IL-1β: interleukin-1β, IL-6: interleukin-6. Created with Biorender.com

Atherosclerosis is a chronic inflammatory condition, resulting from an imbalance in lipid metabolism, vascular function, and a dysfunctional immune response [72]. Extensive basic cellular and animal data strongly support that the immune checkpoint proteins targeted in ICI therapies (CTLA-4, PD-1, PD-L1, and LAG-3) serve as critical negative regulators of atherosclerosis [13, 36, 46]. Blocking these immune checkpoints may accelerate atherosclerosis by enhancing effector T-cell responses, limiting T_reg_ cell function, and increasing immune cell infiltration into the vascular endothelium [13, 46, 68, 82]. It is hypothesized that ICIs may accelerate atherosclerosis and increase the risk of atherosclerotic cardiovascular disease (ASCVD) by enhancing inflammation and immune activation (Fig. 4). In accordance with this, in the study by *Calabretta *et al*.,* routine 18F-FDG PET/CT images were evaluated in 20 ICI-treated melanoma patients to quantify atherosclerotic inflammatory activity. Over a 4-month follow-up period, FDG uptake significantly increased in non-calcified and mildly calcified segments after ICI therapy [16]. This study suggests that ICI therapy could induce low-grade inflammation in the arterial wall and potentially accelerate atherosclerosis progression in treated patients, which might contribute to increased occurrence of cardiovascular events.

Understanding the role of immune checkpoints and immune dysregulation in atherosclerosis is crucial in managing cardiovascular risk in patients treated with ICIs, as well as identifying potential targets for cardiovascular disease beyond the standard of care management.

#### Role of immune cells in atherosclerosis development

Monocyte-derived cells are recruited into the sub-endothelial space, where they differentiate into two activated macrophage phenotypes, M1 and M2. Activated M1 macrophages promote the accumulation of intracellular lipids, initiate and sustain inflammation, and secrete pro-inflammatory factors such as IL-1, IL-6, and TNF-α. In contrast, activated M2 macrophages work toward resolving inflammation, promote the clearance of lipids, and secrete anti-inflammatory factors such as IL-10 and collagen [10, 70, 85].

Apart from monocyte-derived cells, plaque development involves both B and T lymphocytes. However, atherosclerosis is primarily considered a T-cell-driven disease [61, 93, 102]. The molecular characteristics of immune cells within atherosclerotic lesions have been revealed through single-cell analyses [21, 22, 73, 118, 119]. Activated T cells, specifically CD8^+^ cytotoxic T cells and CD4^+^ Th1 cells, are dominantly found in plaques and are associated with proatherogenic cytokine production, like IFNγ and TNFα [36, 39, 47]. Th1 inhibition has shown atheroprotective effects by reducing IFNγ levels in mouse models [65]. In addition, T_reg_ cells play an atheroprotective role through the secretion of anti-inflammatory factors (TGF-β and IL-10) [11, 48], and expression of CTLA-4, which inversely correlates with plaque vulnerability [2, 24, 74]. The roles of other T-cell subsets in atherosclerosis remain less clear. Immune checkpoint inhibitors may activate T cells within plaques, potentially exacerbating atherosclerosis.

#### Immune checkpoints in atherosclerosis

Immune checkpoint proteins are crucial in atherogenesis and have been extensively studied for therapeutic targeting [114]. Basic research models have shown that the PD-1-PD-L1 and CTLA-4 pathways diminish T-cell-driven inflammation, thereby reducing the development and progression of atherosclerotic plaques. Thus, inhibition of these pathways through ICI therapies may lead to T-cell activation within plaques, potentially worsening atherosclerosis in these patients. However, not all inhibited immune checkpoints are likely to enhance atherosclerosis progression; in contrast, inhibition of some may be preventive, such as the macrophage-mediated immune checkpoint CD47 [1, 56, 57].

The PD-1/PD-L1 pathway is crucial in downregulating proatherogenic T-cell responses and mitigating atherosclerosis by restricting APC-dependent T-cell activation. The expression of PD-1 and PD-L1 was significantly downregulated in human PBMCs, T cells, and myeloid dendritic cells in patients with coronary artery disease compared to healthy controls, and reduced levels of PD-1 and PD-L1 were associated with an increased burden of coronary atherosclerotic plaques [68]. *Gotsman *et al*.* demonstrated that PD-L1/2 deficiency in hypercholesterolemic mice is associated with an increased aortic atherosclerotic burden, and higher numbers of CD4^+^ and CD8^+^ T cells in the lesions [46]. Similarly, another study found that mice lacking PD-L1/2 developed larger atherosclerotic lesions enriched with CD8^+^ T cells and macrophages [13]. These studies suggested that PD-L1/2 deficiency resulted in an activated T-cell phenotype with elevated levels of pro-atherosclerotic cytokines, such as IFNγ and TNFα [13, 46]. On the other hand, stimulation of the PD-1/PD-L1 pathway via agonistic PD-1 antibody inhibited the development of atherosclerosis in Ldlr^−/−^ mice with Western diet [20].

Moreover, the role of CTLA-4 signaling has also been investigated extensively in atherosclerotic plaque development. Constitutive overexpression of CTLA-4 in T cells of Apoe^−/−^ mice was found to reduce the formation of atherosclerotic lesions and the accumulation of macrophages and CD4^+^ T cells within plaques [82]. *Poels *et al*.* demonstrated that inhibiting CTLA-4 with monoclonal antibodies in Ldlr^−/−^ mice primarily induced T-cell-driven endothelial inflammation, resulting in increased plaque size [96]. Similarly, in hyperlipidemic mice, combined immune checkpoint blockade with anti-PD-1 and anti-CTLA-4 resulted in increased endothelial activation and the upregulation of vascular adhesion molecules with a higher presence of CD4^+^ and CD8^+^ T cells, and an increase in necrotic core size within plaques, although the overall plaque size remained unchanged [95]. In hypercholesterolemic ApoE3*Leiden mice, co-stimulation blockade with the CTLA-4 fusion protein abatacept attenuated atherosclerosis development via reducing T-cell activation, decreasing circulating IFNγ and increasing IL-10 levels [34].

Moreover, inhibition of LAG-3, a novel immune checkpoint targeted by ICI therapy, has also been associated with atherosclerotic plaque inflammation. *Mulholland *et al*.* showed that in hyperlipidemic mice, inhibition of LAG-3, alone or in combination with PD-1, increased the density of T cells in atherosclerotic plaques, although it did not affect overall plaque size [87].

## Immune checkpoint inhibitors and cardiac arrhythmias

### Clinical relevance

Besides the excess risk of fulminant myocarditis, heart failure, and atherosclerotic cardiovascular disease, ICIs have also been associated with new-onset arrhythmias or cardiac conduction disease. The notion for these side effects was primarily derived from case reports, in which prolongation of the PR intervals, followed by complete atrioventricular block was observed in patients presenting with fulminant myocarditis [58, 60] or myositis [14]. On the other hand, another case report showed that the use of pembrolizumab was associated with slow bidirectional ventricular tachycardia, again in the context of myocarditis [3]. Later, a pharmacovigilance investigation showed a significantly higher reporting signal on new-onset supraventricular arrhythmias in patients receiving ICIs, which were associated with other concurrent irAEs [105]. These findings were supported by a retrospective observational study including 30 patients with established ICI-related cardiotoxicity, pointing out that new-onset atrial fibrillation occurred in 30%, ventricular arrhythmias occurred in 27%, and conduction system disorders were observed in 17% of cases [33]. In this study, the event rates for arrhythmias when no left ventricular systolic dysfunction was present were 3%, 7%, and 13% of patients, respectively, but most importantly, conduction disorders were associated with increased cardiovascular mortality in these patients.

In another registry, electrocardiographic characteristics of patients receiving ICI were compared between patients who developed myocarditis vs. those who did not [127]. Here, as opposed to the above observations, no difference in PR intervals was seen between the groups. Nevertheless, a significantly increased QRS prolongation was present, paralleled by a significant increase in major adverse cardiac events, in patients with vs. without myocarditis. Conversely, QTc did not differ between these cohorts, and it was not associated with the endpoints.

In a Danish nationwide study, significantly higher rates of arrhythmias were also observed in patients with lung cancer or malignant melanoma receiving ICIs compared to patients receiving other anti-cancer medications [30]. In addition, a systematic review of case reports and trial/registry data reported that 10% of ICI-related cardiotoxicity events were attributed to conduction disease, which led to death in 50% of patients [50, 84].

However, contrary to the above findings, a comprehensive meta-analysis of 48 randomized controlled trials showed no increased risk of new-onset supraventricular-, or ventricular arrhythmias, QT-prolongations, or conduction blocks in cancer patients with vs. without ICI treatment [25]. Of note, however, in this analysis, arrhythmias (and all cardiovascular adverse events) were defined using independent MedDRA terms, hinting that events for isolated arrhythmias were obtained, that is, without concurrent myocarditis or heart failure.

Overall, clinical evidence has linked the use of ICIs to an increased risk of new-onset cardiac arrhythmias or conduction disease, which mostly occur secondary to irAEs, e.g., myocarditis, and are less likely to present in an isolated manner. To screen for, and to prevent these side effects is of paramount importance, as complete heart block (i.e., third-degree atrioventricular block) and ventricular tachyarrhythmias after ICI therapy are life-threatening adverse events [78].

### Potential mechanisms

Regarding the effects of ICIs on the cardiac conduction system, experimental studies have observed electrocardiographic abnormalities, including sinus arrest and delayed atrioventricular conduction [120, 125]. However, details on the mechanisms for these effects remain to be elucidated.

ICI-related cardiac adverse effects, such as myocarditis, heart failure, or myocardial infarction, may lead to anatomical remodeling of the cardiac chambers, serving as a substrate for triggered activity, autonomic activity, or re-entrant activity, resulting in supraventricular or ventricular tachycardia [19]. In this scenario, myocarditis and myocardial infarction may lead to the formation of fibrotic tissue as a result of an inflammatory process. In other words, focal fibrotic tissues may favor ectopic activity, leading to re-entrant tachycardia either in the atria (causing atrial flutter/fibrillation/tachycardia) or in the ventricles (leading to ventricular tachycardia/fibrillation) [37]. Nevertheless, an inflammatory state in general (e.g., without the specific involvement of the cardiac tissue) is also a classified substrate for tachycardia, as inflammation facilitates the spontaneous release of sarcoplasmic reticulum calcium, resulting in pro-arrhythmogenic intracellular calcium fluctuation in the cardiomyocytes [66]. In addition, non-inflammatory left ventricular dysfunction, e.g., cardiac chamber dilation or heart failure, also facilitates arrhythmogenicity [19].

Anatomical remodeling of the heart due to ICI-related cardiac side effects may also affect the cardiac pacemaker and conduction system, i.e., the sinoatrial node, atrioventricular node, and the His-Purkinje system. In this case, when fibrosis occurs in the atrioventricular node, PR prolongation, followed by increasing degrees of atrioventricular blocks may occur, resulting in complete heart block in fatal cases.

On the other hand, interactions between leukocytes and the cardiac conduction system have also emerged in the past decade. A seminal basic research study by *Hulsmans and colleagues* showed that macrophage-specific ablation of connexin-43, a gap junction molecule connecting macrophages to cardiomyocytes, results in atrioventricular conduction delay, and ablation of CD11b leads to progressive atrioventricular block [54]. Overall, macrophages are involved in normal and aberrant cardiac conduction, which may be affected by modulation of the immune system. Electrical remodeling has also been linked to inflammation in the context of heart failure with sinus node dysfunction in a recent study by *Kahnert and colleagues* [59]. Here, the research group showed that suppression of the pro-inflammatory galectin-3 molecule prevented sinus node dysfunction in a mouse model of heart failure, also highlighting the significant interaction between inflammation and the cardiac pacing system.

Recently the role of autoantibodies in arrhythmogenesis has also been proposed [71]. Moreover, in a preclinical model of mice with PD-1 deficiency, dilated cardiomyopathy has been associated with anti-cTnI autoantibody formation [88, 89]; however, further research is needed to elucidate the role of autoantibodies related to immune checkpoint deficiency or blockade in arrhythmogenesis.

**Fig. 5:**
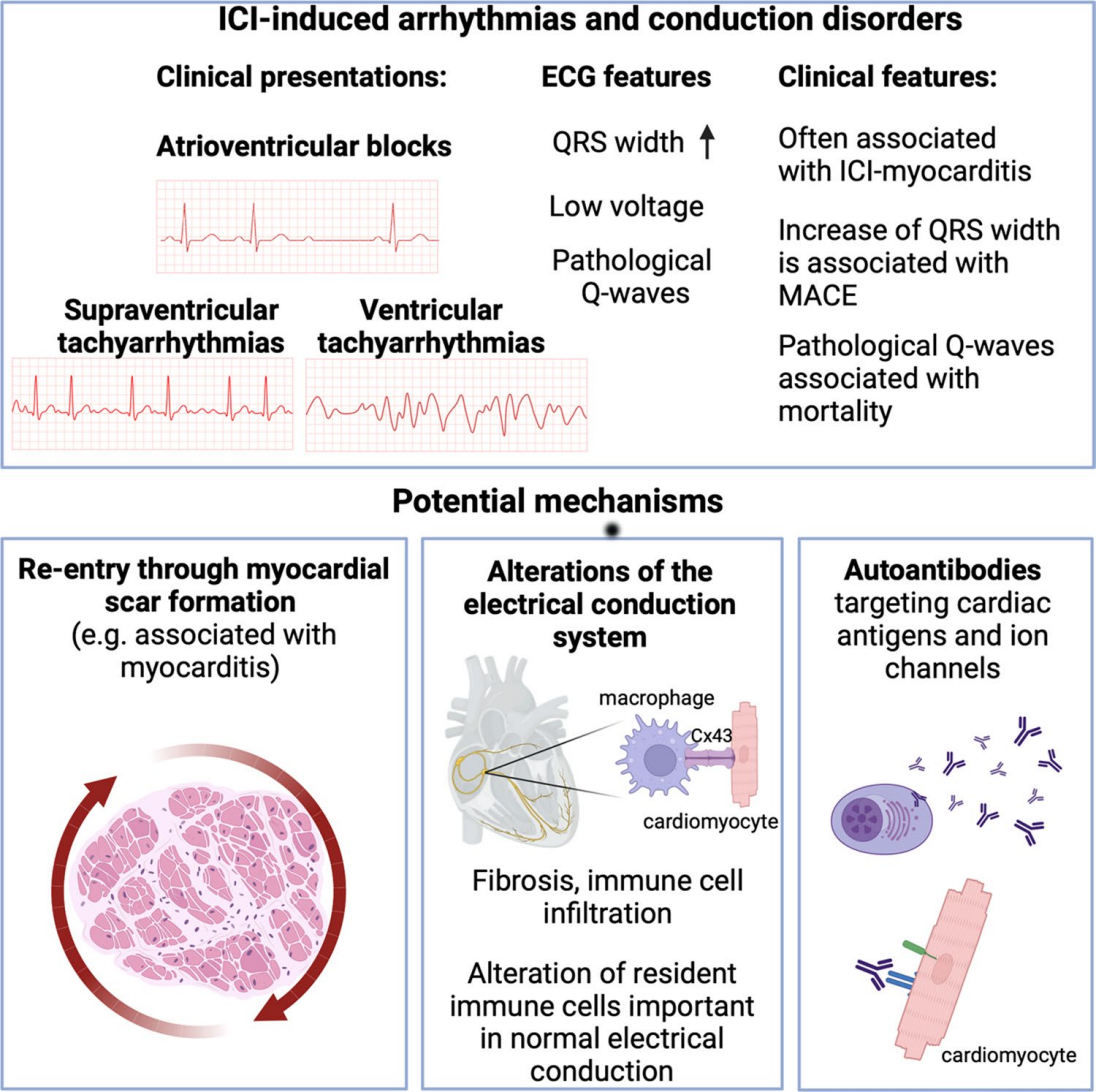
Immune checkpoint inhibitor-induced arrhythmias and conduction disorders. ICI-induced electrophysiological disorders are often associated with ICI myocarditis and include atrioventricular blocks, as well as supraventricular and ventricular tachyarrhythmias. Potential, hypothetical mechanisms include re-entry through myocardial scar formation, alteration of immune cells (e.g., macrophages) important in physiological electrical conduction in the myocardium, and autoantibody formation, although mechanistic studies are needed to confirm these hypotheses. MACE: major adverse cardiovascular events. Created with Biorender.com

Overall, mechanisms for ICI-related arrhythmias or conduction diseases are largely unknown currently. Nevertheless, it is suggested that these side effects are likely attributed to anatomical remodeling of the heart, as ICI-related dysrhythmias are mostly observed in the context of other irAEs, and less frequently in an isolated manner (Fig. 5). However, currently unknown mechanisms may also underlie these relations, urging for further investigations in both the preclinical and the clinical arena.

## Immune checkpoint inhibitors and vasculitis

### Clinical relevance

Vasculitis has been reported in connection with immune activation resulting from immune checkpoint inhibitors (ICIs), with large vessel vasculitis being more common [12]. A large retrospective pharmacovigilance study of the World Health Organization’s Global Database for Case Safety identified an increased incidence of giant cell arteritis following ICI therapy, especially after CTLA-4-targeted therapy. The median time to vasculitis onset was 55 days (ranging from 21 to 98 days) after initiating ICI therapy, with a mortality rate of 6% (5/82) [105]. Similarly, in a pharmacovigilance study, CTLA-4, but not PD-1/PD-L1 inhibition, was associated with over-reporting of giant cell arteritis [101]. A scoping review of 24 publications examined 29 ICI-associated vasculitis cases [67]. The mean time to symptoms onset from the start of ICI was 7.2 months, with over half of the cases occurring more than 3 months after beginning the ICI treatment. Vasculitis treatment predominantly involved glucocorticoids in 96% of cases, and immunotherapy was frequently discontinued (44.8%). Data indicate that vasculitis generally has a delayed onset after starting immunotherapy, and the outcomes are typically positive, with responses to glucocorticoids and the discontinuation of immunotherapy [67].

### Potential mechanisms

The pathophysiological mechanisms of ICI-related large vessel vasculitis are not well understood. T cells and macrophages infiltrate all layers of the vessel wall, whereas dendritic cells embedded in the vessel wall, expressing CD80, CD86, PD-L1, and PD-L2, play an important role in preventing immune cell infiltration. High expression of PD-L1 on dendritic cells has been associated with maintaining healthy vasculature [15]. In giant cell arteritis, the balance between co-stimulatory signals (such as CD28) and co-inhibitory (such as CTLA-4 and PD-1) determines the level of inflammatory activity within the blood vessel walls [15]. Vascular infiltrating CD4^+^ T cells produce cytokines like IFNγ, IL-9, IL-17, and IL-21, which contribute to intimal hyperplasia and intramural neoangiogenesis [75, 94]. In addition, during active vasculitis, macrophages produce various cytokines, including TGF-β, IL-1β, and IL-6, particularly within the adventitia [109].

In a study by *Régnier *et al*.,* arterial tissues from patients with giant cell arteritis showed increased expression of CTLA-4 but not PD-1, compared to control arteries [101]. Here, the authors also showed via transcriptomic data that CTLA-4-related pathways are upregulated in the circulating CD4^+^ T-cell compartment of patients with giant cell arteritis, while in aortic samples, gene expression of CD4^+^ T cells was also altered. Overall, T_regs_ were decreased in number and responsiveness in GCA patients, while the remaining T_reg_ population overexpressed CTLA-4 as a compensatory mechanism. Inhibition of CTLA-4 by ipilimumab in vitro led to increased depletion in PBMCs from GCA patients compared to controls. These findings may also correlate with the fact that T-cell co-stimulation blockade with abatacept reduces the risk of relapse of GCA [63].

Furthermore, transcriptome data from temporal artery biopsies in patients with giant cell arteritis revealed low levels of PD-L1 in vessel wall dendritic cells, alongside relatively high levels of PD-1 receptors in T cells [124]. Inhibition of the PD-1 pathway resulted in unchecked T-cell activation, leading to the release of cytokines that promote vasculitis.

## Immune checkpoint inhibitors and thromboembolism

### Clinical relevance

In a retrospective cohort study of 2,854 patients, the incidence of venous thromboembolism (VTE) was found to be notably high and increased after initiating ICI therapy [43]. The incidence of VTE was 7.4% at 6 months and 13.8% at 1 year after starting an ICI. The hazard ratio (HR) for deep vein thrombosis was 5.70 (95% CI 3.79–8.59), and for pulmonary embolism, it was 4.75 (95% CI 3.20–7.10) [43]. A systematic review and meta-analysis of 16,602 patients with non-small cell lung cancer treated with ICIs reported thromboembolism rates ranging from 0.1% to 13.8%. The pooled rate of high-grade thromboembolisms was 1% (95% CI 1%–2%). The VTE rate was 3% (95% CI 2%–4%), and the arterial thromboembolism rate was 1% (95% CI 1%–2%). Patients receiving a combination of immunotherapy and chemoradiotherapy exhibited the highest incidence of thromboembolisms at 7%. The pooled thromboembolism rate was higher in patients treated with combined ICIs than those treated with monotherapy ICIs (4% vs. 2%) [122].

### Potential mechanisms

The mechanisms underlying thrombotic events following ICI therapy are not understood due to insufficient evidence. Inflammatory processes have been linked with venous thromboembolism by activating the coagulation cascade and platelets [32, 38]. It has been hypothesized that the incidence of thromboembolism after immune checkpoint inhibition may be linked to the activation of T cells, which in turn release pro-inflammatory cytokines (e.g., IL-1β, IL-6, TNFα) and activate monocytes/macrophages. Activated monocytes/macrophages show increased tissue factor expression leading to the activation of the coagulation cascade, promoting hypercoagulability [52, 106].

## Immune checkpoint inhibitors and pericarditis

### Clinical relevance

The occurrence of pericarditis as an irAE may progress slowly and without clear symptoms, and the timing of onset may vary. A systematic review of the published ICI-associated pericardial disease included 20 publications, comprising a total of 28 cases. ICI-associated pericardial disease was reversible in 75% of the cases, with a 7% mortality rate [55]. In a retrospective cohort study, *Gong *et al*.* found a fourfold increase in the risk of pericarditis or pericardial effusion among patients treated with ICIs compared with controls not treated with ICIs, after adjusting for potential confounders (HR 4.37, 95% CI 2.09–9.14). The incidence rate was 1.57 events per 100 person-years in the ICI group [44].

### Potential mechanisms

The mechanisms behind immune-mediated pericardial damage can be complex and multifactorial. Clinical observations and preclinical studies have proposed several hypotheses regarding the development of cardiotoxicity secondary to ICI therapy. These include targeting of shared antigens by both tumors and homologous heart tissue by clonally expanded T cells, and antitumor activity in malignant pericardial or myocardial involvement cases [6]. In a case series on pericarditis, several potential mechanisms were suggested. Reduced granzyme B expression in pericardium-infiltrating T cells indicates a mechanism distinct from granule exocytosis. However, cytotoxic T cells may still contribute to toxicity through cytokines such as FasL and TRAIL. Increased CD68^+^ expression in pericarditis samples raises the question of whether PD-1/PD-L1-targeted treatments disrupt macrophage function, resulting in heightened organ-specific activity [6].

## Immune checkpoint inhibitors and dyslipidemia

### Clinical relevance

In the meta-analysis by *Dolladille *et al*.* investigating 48 RCTs with ICI therapy, dyslipidemia was associated with the highest incidence after ICI treatment among the cardiovascular adverse effects (19.3 (6.7–54.1) for 1000 patients; Peto OR: 3.68, 95% CI 1.89–7.19, *P* < 0.01). As discussed previously, ICI therapy is associated with increased plaque progression through enhancing plaque inflammation; however, these findings suggest that increased occurrence of dyslipidemias may also contribute to the development of ASCVD. Further research is needed to determine the association between lipid levels and ICIs, whereas the role of lipid-lowering therapy in ICI-treated patients must also be elucidated.

### Potential mechanisms

Interestingly, in one preclinical study using Ldlr^−/−^ Pdcd^−/−^ mice, not only atherosclerotic plaque size but also serum lipid levels were found to be higher compared to Ldlr^−/−^ mice, suggesting that PD-1 deficiency further aggravates dyslipidemia [20], although in another preclinical study using hypercholesterolemic mice, serum lipid levels did not differ [13]. In addition, increased cholesterol in the tumor microenvironment induces T cell exhaustion, suggesting a link between cholesterol levels and immune checkpoint expression on T cells [80].

## Potential common mechanisms of ICI-related cardiovascular toxicities

While our understanding of the mechanism of some ICI toxicities is quite advanced, e.g., in the case of myocarditis or atherosclerosis, we have less data available about the mechanisms of ICI-related arrhythmias, heart failure, vasculitis, pericardial disease, or venous thromboembolism. Nevertheless, some common mechanistic patterns arise within the spectrum of ICI cardiovascular toxicity. First, T-cell activation by immune checkpoint blockade leads to pro-inflammatory cytokine production, which was observed in the majority of adverse effects, including increased levels of IL-1β, IL-6, IL-17, IFNγ, and TNFα, whereas the cellular communication between T cells and macrophages seems to play a vital role as well in mediating the cardiovascular effects of ICIs. Moreover, the thymus has emerged as a potential central mediator of ICI cardiotoxicity, as it has been associated with myocarditis in a clinical study [35], while in preclinical experiments, alterations of pro-inflammatory signaling in the thymus have been found in animals with cardiac dysfunction [41]. Understanding the common mechanisms between ICI-related cardiovascular adverse events may pave the way for broader use of cardioprotective therapies against ICI cardiotoxicity; however, the effects of cardioprotective strategies on cancer growth and oncological outcomes should also be considered, as these may often counteract the desired anti-cancer effects [51].

## Conclusion

Our comprehension of the impacts of various immunotherapeutic approaches on cardiovascular structure, function, and major cardiovascular events is in its early stages, evolving, and still relatively limited. It is imperative to conduct rigorous preclinical and clinical studies to enhance our understanding of the epidemiological relevance and pathomechanisms of these side effects. Moreover, it is important to acknowledge how these cancer treatments can offer unique insights into cardiovascular biology, providing actionable information applicable to both cancer and non-cancer patients.

ICIs are now strongly linked with cardiovascular toxicities such as myocarditis, heart failure, arrhythmias, pericardial disease, atherosclerosis, thromboembolism, and vasculitis. Some of these cardiovascular immune-related adverse effects necessitate prompt immunosuppressive intervention and even some times discontinuation of ICIs, especially due to their high fatality rates. With nearly 600,000 eligible ICI therapy candidates in the USA alone [116], and with the anticipated increase in ICI usage in the future, it is crucial to understand the pathomechanisms of these side effects to identify common therapeutic targets for managing the spectrum of cardiovascular toxicities. Achieving this requires closer collaboration among cardiology, oncology, and immunology in both clinical practice and basic research to better recognize and manage the cardiovascular toxicities associated with ICI therapies.
